# Supplementary material for: Vitis OneGenE: A Causality-Based Approach to Generate Gene Networks in Vitis vinifera Sheds Light on the Laccase and Dirigent Gene Families
Source: Biomolecules. 2021 Nov 23;11(12):1744. doi: 10.3390/biom11121744 (PMC8698957; doi:10.3390/biom11121744)
Supplement: Supplementary file 1 [file biomolecules-11-01744-s001.zip › biomolecules - 1435143 supp/Figure S1.pdf]

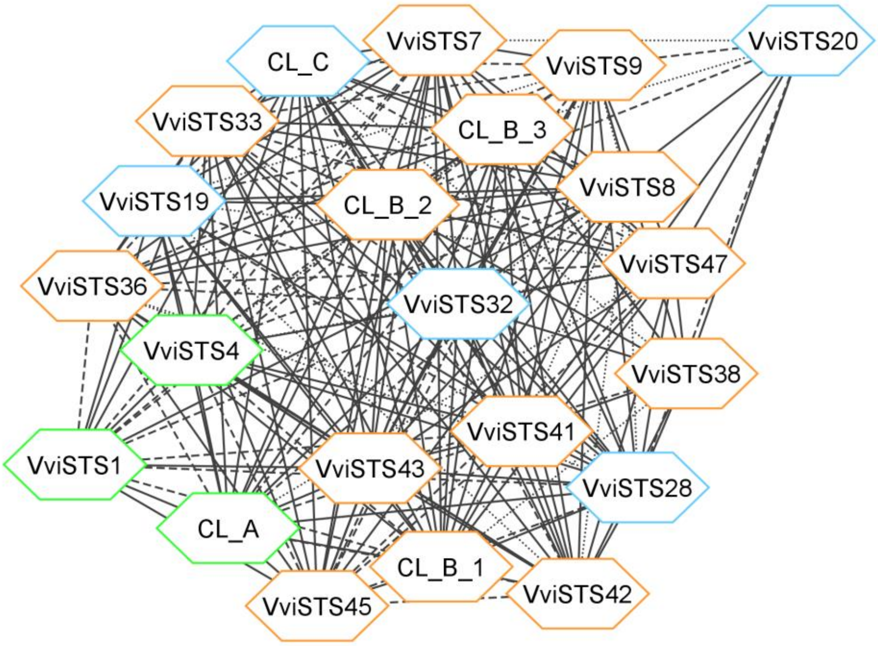

Figure S1: The Stilbene synthase (STS) network. Nodes are named as explained in Table S1. Node border colors (green, orange, light blue) correspond to the *VviSTS* phylogenetic groups A, B and C, respectively. Edge line types (solid, dashed or dotted) are used to represent edge weight, based on the mean relative frequency (from 1 to 0.5).
